# Supplementary material for: Stool Banking for Fecal Microbiota Transplantation: Methods and Operations at a Large Stool Bank
Source: Front Cell Infect Microbiol. 2021 Apr 15;11:622949. doi: 10.3389/fcimb.2021.622949 (PMC8082449; doi:10.3389/fcimb.2021.622949)
Supplement: Supplementary file 2 [file DataSheet_2.pdf]

Supplemental Information for Chen *et al.* “Stool banking for fecal microbiota transplantation: methods and operations at a large stool bank” – Online health questionnaire

Preamble

Please answer all questions of this survey completely to be considered for the next phase of the application process. Being completely honest in your answer, to the best of your ability, is very important to patient safety.

By participating in this survey, you are agreeing to allow the OpenBiome clinical team to use the information you provide to help determine your eligibility as a stool donor. No one outside the clinical team will be able to connect your responses with any information that identifies you, such as your name, date of birth, email address, and other contact information. If you do not qualify for the stool donation program or choose not to participate, we will not keep any of your identifying information.

Questions

- Name
- Contact information (email address, phone number)
- Date of birth
- Gender
- Height, weight, and body mass index
- Will you be living or working in the greater Boston area for the next six months? (Yes/No)
- ZIP Code (home and work)
- How many times per week are you able to donate stool at our [address redacted] location? We are open [hours redacted].
- Have you applied to be a donor for OpenBiome before? (Yes/No)
- Have you, or an immediate relative (brother, sister, parent, or child), had colon cancer? (Yes/No)
- Have you, or an immediate relative (brother, sister, parent, or child), had Inflammatory Bowel Disease (i.e. Crohn's Disease or Ulcerative Colitis)? (Yes/No)
- In the past 12 months, have you been treated by a doctor for asthma or had asthma symptoms? (Yes/No)
- Do you regularly experience fever or any stomach issues, such as constipation, diarrhea, stomach pain, or bloating? (Yes/No)
- In the last 12 months, have you had treatment, taken medication, or attended counseling for Attention Deficit Disorder (ADD) or Attention Deficit Hyperactivity Disorder (ADHD)? (Yes/No)
- In the last 12 months, have you had treatment, taken medication, or attended counseling for depression? (Yes/No)
- In the last 12 months, have you regularly experienced symptoms of depression? (Yes/No)
- In the last 12 months, have you had treatment, taken medication, or attended counseling for anxiety? (Yes/No)
- In the last 12 months, have you regularly experienced symptoms of anxiety? (Yes/No)

- Do you have any of the following allergies? Please select all that apply. (No known allergies; Seasonal; Food; Pet; Medication; Latex; Dust; Other)
- In the last 8 weeks, have you experienced symptoms of eczema or psoriasis? (Yes/No)
- In the last 3 months, have you had unprotected sex with a new sexual partner? (Yes/No)
- In the last 6 months, did you get a new tattoo? (Yes/No)
- In the last 6 months, did you get a new piercing? (Yes/No)
- In the last 8 weeks, have you taken any antibiotics, antifungals, or antivirals? (Yes/No)
- Does your work or any volunteer activity involve contact with any of the following? Hospitals; Long-term care settings [e.g. nursing homes]; Outpatient setting [e.g. medical clinic, physical therapy, or dialysis]; Human or animal tissue. (Yes/No)
- In the last 8 weeks, have you had any of the following shots or vaccinations? Please select all that apply. (I have not had any shots in the last 8 weeks; Adenovirus; Anthrax; Cholera; DTaP; DT; Hepatitis A; Hepatitis B; Herpes Zoster [shingles]; HPV; Influenza; Japanese encephalitis; Measles, Mumps, Rubella [MMR]; Measles, Mumps, Rubella, Varicella [MMRV]; Meningococcal; Pneumococcal; Polio; Rabies; Rotavirus; Tetanus; Typhoid; Varicella; Vaccinia [smallpox]; Yellow Fever)
- Select all of the countries you have visited in the last 12 months. (I have not traveled outside of the US in the past 12 months; list of all world countries)
